# Integrative effects of phytohormones in the phenolic acids production in *Salvia verticillata* L. under multi-walled carbon nanotubes and methyl jasmonate elicitation

**DOI:** 10.1186/s12870-023-04719-5

**Published:** 2024-01-19

**Authors:** Nosrat Rahmani, Tayebeh Radjabian

**Affiliations:** Department of Biology, Faculty of Basic Sciences, Shahed University, Tehran, Iran

**Keywords:** Methyl jasmonate, Multi-walled carbon nanotubes, Phenolic acids, Phytohormones, *Salvia verticillata* L.

## Abstract

*Salvia verticillata* L. is a well-known herb rich in rosmarinic acid (RA) and with therapeutic values. To better understand the possible roles of phytohormones in the production of phenolic acids in *S. verticillata*, in this work, we investigated some physiological and biochemical responses of the species to methyl jasmonate (MJ) and multi-walled carbon nanotubes (MWCNTs) as two effective elicitors. The leaves were sprayed with aqueous solutions containing 100 mg L^−1^ MWCNTs and 100 µM MJ and then harvested during interval times of exposure up to 96 h. The level of abscisic acid, as the first effective phytohormone, was altered in the leaves in response to MJ and MWCNTs elicitation (2.26- and 3.06-fold more than the control, respectively), followed by significant increases (*P ˂* 0.05) detected in jasmonic acid and salicylic acid contents up to 8 h after exposure. Obtained data revealed that simultaneously with changes in phytohormone profiles, significant (*P ˂* 0.05) rises were observed in the content of H_2_O_2_ (8.85- and 9.74-folds of control), and the amount of lipid peroxidation (10.18- and 17.01-folds of control) during the initial times after exposure to MJ and MWCNTs, respectively. Later, the content of phenolic acids increased in the elicited leaves due to changes in the transcription levels of key enzymes involved in their biosynthesis pathways, so 2.71- and 11.52-fold enhances observed in the RA content of the leaves after exposure to MJ and MWCNTs, respectively. It is reasonable to conclude that putative linkages between changes in some phytohormone pools lead to the accumulation of phenolic acids in the leaves of *S. verticillata* under elicitation. Overall, the current findings help us improve our understanding of the signal transduction pathways of the applied stimuli that led to enhanced secondary metabolite production in medicinal plants.

## Background

*Salvia verticillata* L., popularly known as lilac sage or purple rain, belongs to the Lamiaceae family. It is a perennial herbaceous plant native to the Mediterranean, Central Asia, and Western China [1, 2]. Because of its anti-inflammatory, antibacterial, and antioxidant characteristics, *S. verticillata* has long been used medicinally [3]. This plant is packed with a wide variety of phytopharmaceuticals that possess a range of medicinal benefits. These bioactive compounds comprise fatty acids, alkaloids, terpenoids, phytosterols, phenolic compounds (specifically phenolic acids), and flavonoids [4–7].

Phenolic acids are a type of secondary metabolites that possess remarkable antioxidant and anti-inflammatory properties [8]. These compounds function to improve plant growth and development while providing adequate protection against various environmental stressors like infections, herbivores, and UV radiation [9]. A screening project on several *Salvia* species demonstrated that *S. verticillata* is a rich source of phenolic acids, especially rosmarinic acid (RA) [10, 11]. RA and its derivatives, such as salvianolic acids, are thought to be responsible for a wide range of biological and pharmacological effects in plants that contain these compounds. RA has anti-inflammatory potential, and studies indicate that it may aid in the cure of inflammatory diseases like arthritis, asthma, and atopic dermatitis [12]. This phenolic acid is also extensively utilized in the food industry as a natural preservative due to its antioxidant properties. It has also been studied for its possible therapeutic uses in a variety of disorders, including diabetes [13], Alzheimer’s disease [14], and some cancers [15].

RA and its derivatives are co-produced through the phenylpropanoid and tyrosine-derived pathways. Phenylalanine ammonia-lyase (PAL) and tyrosine aminotransferase (TAT) serve as the critical initial enzymes in their respective paths. In addition, rosmarinic acid synthase (RAS) connects the two compounds of the parallel pathways, 3,4-dihydroxyphenylacetic acid and caffeine-CoA, to synthesize RA. It is possible to convert the RA molecule to lithospermic acid and salvianolic acids as derivatives of RA. The steps of these reactions are not yet fully understood [16–18].

The control of phenolic acid production in plants is complicated, including several routes and variables. Environmental factors like nanomaterial pollutants [19, 20], light [21], temperature [22], and water availability [23] all could have a meaningful impact on phenolic acid synthesis [24]. Furthermore, phytohormones such as auxins, cytokinins, salicylic acid (SA), jasmonic acid (JA), cytokinins, and abscisic acid (ABA) might play an imperative role in regulating their production [25]. These messengers can trigger signaling pathways, resulting in the activation of genes implicated in the phenylpropanoid pathway, which is responsible for the production of phenolic acids [26–28].

Multi-walled carbon nanotubes possess exceptional properties that have attracted significant attention across various sectors, making them highly desirable nanomaterials. MWCNTs have the potential significantly affect different aspects of plant life, including growth, development, metabolism, physiology, biochemistry, and even gene expression patterns [29–35]. When plants are exposed to MWCNTs, it can cause oxidative stress leading to the production of reactive oxygen species (ROS) and lipid peroxidation. It can be harmful to plants if this happens. Studies have revealed that *Catharanthus roseus* (L.) G.Don [36], *Salvia nemorosa* L. [37]. , *Satureja Khuzistanica* Jamzad [38], and *Thymus daenensis* Celak [39] tend to produce and accumulate phenolic acids when exposed to MWCNTs. It is well known that MWCNTs can affect plants by activating stress-related pathways and regulating genes responsible for phenolic acid production [38].

Methyl jasmonate, the methyl ester derivative of JA, is widely used as a stimulating agent in a range of in vitro and in vivo studies. Plants utilize MJ as a signaling molecule to trigger their defense mechanisms in response to biotic stresses [40]. The effectiveness of exogenous MJ in triggering plant defense mechanisms has been confirmed through various studies, resulting in a significant increase in secondary metabolite production. The exposure of plants to MJ triggers cascade reactions that activate the induction of genes responsible for the biosynthesis of secondary metabolites [40, 41]. Consequently, numerous researchers have employed MJ as an effective elicitor to enhance the production of secondary metabolites in medicinal plants, including phenolic acids [42–45]. Pesaraklu et al. [44] have described that elicitation with MJ significantly enhanced the production of phenolic acids, notably RA, and the transcript of the crucial genes (*TAT*, *PAL*, and *RAS*) in *S. verticillata*. Furthermore, in a previous study, we showed that foliar elicitation of *S. verticillata* with low concentrations of MWCNTs promotes the production of RA through changes in the expression levels of key enzymes involved in the phenolic acid biosynthesis pathway [46]. Although several data describe the effect of different elicitors on phenolic acids biosynthesis, no reports are available dealing with changes in phytohormone profiles. This study focuses on the role of phytohormones in controlling phenolic acid levels in *S. verticillata* under MWCNTs and MJ treatments. It points out how critical it is to comprehend the role of phytohormones in plant responses to these stresses. Understanding the complicated connections between the functions of phytohormones and response pathways to stress leads investigators to develop strategies for improving the production of RA in plants.

## Materials and methods

### Plant materials and treatments

The plants and mature seeds of *Salvia verticillata* were harvested from Alamut Mountain (Qazvin province, Iran) with geographic coordinates 36°20΄58˝ N, 50°46΄57˝ E, and 2250 m of the sea level. The plant voucher specimen was identified by Dr. Masoud Ranjbar and kept at the Bu-Ali Sina University herbarium of with voucher number of BASU 33996. The seeds were germinated in the incubator under dark conditions at 25° C for two weeks. The healthy seedlings were sown in plastic pots covering perlite, soil, and cock peat at 1:3:1 by volume. They were maintained and grown in a phytotron with 60–70% relative humidity and florescent lamps (450 µmol m^−2^ s^−1^ intensity for 16 h per day). The temperature was kept between 22 and 25 °C. After two months, twenty-leaf developmental stage plants were subjected to treatment with elicitors.

The COOH-functionalized MWCNTs (US Research Nanomaterials, USA, US4311, 95%) and MJ (Sigma-Aldrich, 392707, 95%) were applied for cultivated *S. verticillata* treatment. The physicochemical properties of MWCNTs, including SEM images, XRD patterns, Raman, and FTIR spectra, were characterized as described in our previous paper [46]. Plant samples were foliar sprayed with aqueous solutions of 100 mg L^−1^ MWCNTs and 100 µM MJ, and distilled water was used as the control treatment. Because MJ has low solubility in water, it was dissolved in ethanol (0.1%) and diluted with distilled water and a 100 µM stock solution of MJ was prepared [47]. The leaf samples were harvested at 0, 0.5, 1, 2, 4, 8, 12, 24, 48, 72, and 96 h after treatment. Sampling was done in triplicate for each treatment. Half of each plant sample was stored at -80 °C, and half the rest was dried in an oven at 40 °C for further analyses.

### Determination of lipid peroxidation and hydrogen peroxide (H_2_O_2_)

Lipid peroxidation in the treated leaves was estimated by measuring malondialdehyde (MDA) content in the reaction with thiobarbituric acid [48]. The content of MDA was calculated using the extinction coefficient 0.155 µM^−1^cm^−1^ and was expressed as µM g^−1^ FW.

The amount of H_2_O_2_ in the samples was determined by potassium iodide reaction according to Sergiev et al. [49] method with the extinction coefficient (0.28 µM^−1^ cm^−1^), and the results were reported as µM g^−1^ FW.

### Phenolic acids content

#### Extract preparation

The extraction procedure of the main phenolic acids containing RA, salvianolic acid B (Sal B), caffeic acid (CA), and salvianolic acid A (Sal A) in the treated leaves was done according to the method described in our previously published report [46]. The dried and powdered leaf samples (1 g) were homogenized in 5 mL methanol and then incubated at 25 °C for 24 h in the darkness. After that, the mixtures were centrifuged at 12,000 rpm for 10 min, and the obtained supernatants were kept at 4 °C until analysis.

#### HPLC analysis

The phenolic acids were identified in the samples using a Smartline HPLC apparatus (Kenuer GmbH HPLC, Germany) coupled with an ultraviolet detector (UV). The separation was carried out in a C18 MZ-Analysen Technik (250 × 4.6 mm, 5 μm). The mobile phase was acetonitrile (solvent A) and 0.1% (v/v) phosphoric acid in water (solvent B) with gradient elution as follows: 10–25% A (v/v) at 0–15 min, 25–80% A (v/v) at 15–40 min, 80–100% A (v/v) at 40–45 min, 100% A (v/v) at 45–50 min, 100–10% A (v/v) at 50–60 min. A volume of 20 µL of each extract was injected, and the detection method was done at 280 nm. The flow rate of the solvent was 1 mL min^−1^. Data analysis was performed using ChromGate (V 3.1) software. The identification of peaks in the chromatograms was attained by comparison of their retention times with four phenolic acid standards containing RA (Aldrich, 96%), Sal A (Fluka, 95%), Sal B (Fluka, 94%), and CA (Sigma, 98%). The content of each phenolic acid in the treated leaves was computed via the standard calibration curve (y_RA_=41606x, R^2^ = 0.9961; y_Sal B_=20551x, R^2^ = 0.9954; y_Sal A_=19497x, R^2^ = 0.9967; y_CA_=7149x, R^2^ = 0.9931) and represented as mg g^−1^ DW.

### Relative expression pattern of *PAL*, *TAT*, and *RAS* genes

To obtain total RNA, frozen leaves (80–100 mg) were ground in liquid nitrogen with a mortar and pestle. Extraction with RNX-Plus Kit (RN7713C, CinnaGen, Iran) has been applied based on the manufacturer’s orders with minor changes. The purity and concentration of the obtained RNAs were measured using 1.0% agarose gel electrophoresis and SPECTROstar Nano (BMG Labtec, Germany), respectively. First-strand cDNA was synthesized from 1 µg total RNA of each sample using the PrimeScript™ RT Reagent Kit (TaKaRa, Japan), according to the program: 37 °C for 15 min, 85 °C for 5 min. The prepared cDNAs were saved at -80 °C for RT-qPCR.

The relative gene expression patterns of PAL, TAT, and RAS enzymes were calculated using Real-time quantitative PCR (Rotor-Gene 6000, QIAGEN, USA), conducted SYBR Premix Ex TaqII (TaKaRa, Japan). Specifications and sequence of the used primers are shown in Table 1. Real-time conditions were set as 95 °C for 2 min (1 cycle), 40 cycles containing denaturation at 95 °C for 10 s, 30 s at annealing temperature, and 72 °C extensions for 30 s. Melting curve analysis was plotted from 72 to 96 °C. Expression analysis was normalized relative to *rbcL* (ribulose 1, 5-bisphosphate carboxylase/oxygenase large subunit gene) as a reference gene and then calculated with the comparative CT technique described by Livak and Schmittgen [50].

**Table 1:** Specifications of the primers used in this study

Gene	Primers	Sequence (5´→3´)	Annealing temperature (°C)
*SvPAL*	Forward	CATGAGTAAGGGCACCGACAG	62
	Revers	AGTAGCCTTGGAGGAGGGTGTT	
*SvTAT*	Forward	TCAGCACCTGAAAGAAGATTG	57
	Revers	AACCAGGAACCAACCATCTC	
*SvRAS*	Forward	CAGTTGACTCGGTTCAAATGCGG	62
	Revers	TGATGAAGTGGAGAGCGGAG	
*SvrbcL*	Forward	TTGGCAGCATTCCGAGTAAC	57
	Revers	TGGTAAGTCCATCGGTCCAC	

### Phytohormones analysis

#### Sample extraction

Phytohormones were extracted using the method described by Engelberth et al. [51] and Muller et al. [52] with some modifications. Accordingly, leaf samples (2 g) were excellently powdered by a mortar and pestle in liquid nitrogen and next homogenized with 3 mL buffer comprising 2-propanol: water: HCL in a ratio of 2:1:0.002 for 1 min. The obtained homogenates were sonicated in an ultrasonic bath for 30 min at 4 °C. In the next step, 1 mL dichloromethane was added to the homogenates, then vortexed for 1 min and centrifuged at 3000 rpm for 15 min at 4 °C. All lower phases were pooled and transferred into the new glass tubes, and their solvents were evaporated using laboratory nitrogen gas (with a high purity percentage). The precipitates were dissolved in 250 µL of dichloromethane and kept at -20 °C for further analyses.

#### HPLC-EIS-MS/MS

HPLC analysis of the examined phytohormones (GA_3_, ABA, JA, and SA) was carried out using an HPLC-ESI-MS/MS (Waters Alliance 2695) system consisting of a micro-mass Quattro micro-API mass spectrometer. The separation of compounds was performed at 30 °C on an Atlantis T3-C18 column with 3 μm particle size, 100 mm length, and 2.1 mm diameter. The mobile phase comprised 0.1% formic acid in water (50%) and 0.1% formic acid in acetonitrile (50%) with an isocratic elution program. The flow rate of the mobile phase was 0.2 mL min^–1^ throughout the run, and the injection volume for samples was 5.0 µL. We quantified all phytohormones using multiple reaction monitoring (MRM) in negative mode by applying separate retention time windows. The optimal conditions for the ionization source were as follows: ion spray voltage 3500 V, DE temperature 300 °C, source temperature 120 °C, and collision energy 25 eV. Nitrogen was used as curtain gas with a flow rate of 200 L h^−1^. To determine the concentration of phytohormones in the extracts, we used an equation derived from the calibration curves (y_JA_ = 2.312x + 0.1699, R^2^ = 0.9991; y_ABA_ = 1.24256x + 0.99721, R^2^ = 0.9981; yGA_3_ = 3.56112x + 0.169908, R^2^ = 0.9991; y_SA_ = 10.8929 x + 102.371, R^2^ = 0.9974) of standard compounds (JA: Sigma-99%, SA: Merck-99%, ABA: Sigma-99%, and GA_3_: Merck-90%) and represented as ng g^−1^ FW.

### Statistical analyses

The research was carried out as a factorial experiment using a randomized complete block design (RCBD) with three independent replications. The data presented as the mean ± standard errors. The data were analyzed using one-way ANOVA followed by Duncan’s multiple tests for comparing multiple means. The analysis was conducted at a significance level of *P ˂* 0.05 using SPSS software (version 22). The correlations were analyzed (average linkage and Pearson distance metrics) to create a heatmap of relationships between all observed parameters using the open-source software R (https://www.r-project.org/) and RStudio (https://www.rstudio.com/). Furthermore, the principal component analysis (PCA) was applied with the PAST program to reveal the pattern and link between the used treatments and researched parameters (Version 3.15).

## Results

### Effect of MWCNTs and MJ on MDA and H_2_O_2_ contents

**Fig. 1:**
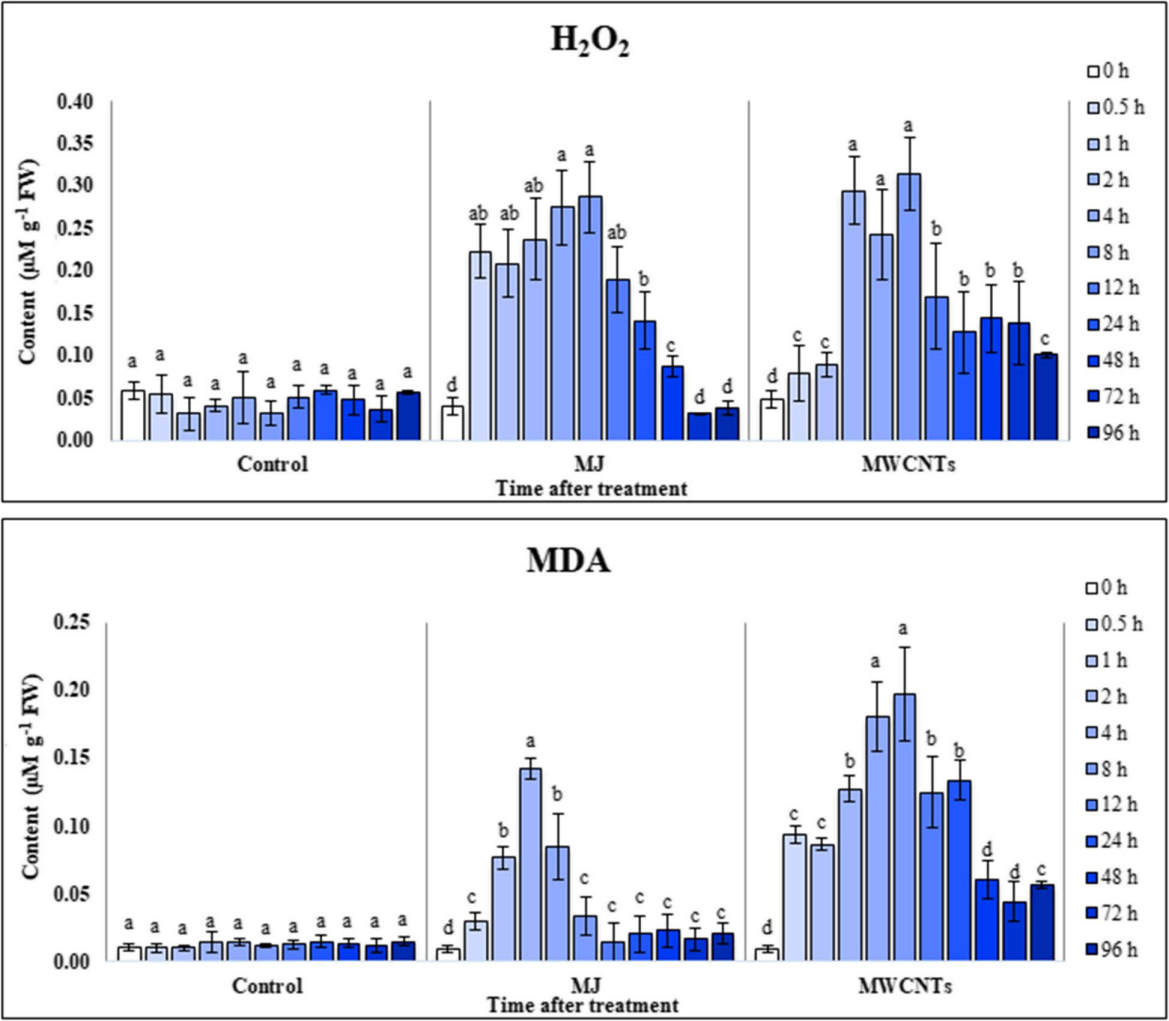
Changes in the malondialdehyde (MDA) and hydrogen peroxide (H_2_O_2_) contents of the *S. verticillata* leaves after elicitation with MWCNTs and MJ. Bars not sharing the same superscript letter(s) are significantly different at *P <* 0.05 within each treatment group based on Duncan’s multiple range test. All data are means of three replicates, with error bars indicating SE

To assess how MJ and MWCNTs affected oxidative stress responses in the treated leaves, we measured the levels of H_2_O_2_ and MDA as indicators of lipid peroxidation. The results depicted in Fig. 1 exhibit a noteworthy dissimilarity (*P ˂* 0.05) in the levels of H_2_O_2_ and MDA between the treated and control plants when both elicitors were employed.

The patterns of changes in H_2_O_2_ levels in the plants treated with MWCNTs were approximately similar to those of plants elicited with MJ. According to the data, H_2_O_2_ generation was increased up to 8 h in the leaves following exposure to MWCNTs (0.31 ± 0.04 µM g^−1^ FW) and MJ (0.28 ± 0.04 µM g^−1^ FW), which was about 9.74 and 8.85- folds higher than those found in control samples (0.03 ± 0.01µM g^−1^ FW), respectively. After that, H_2_O_2_ generation in the treated samples decreased with time.

By measuring lipid peroxidation levels in the treated leaves, we found that the MDA content was significantly (*P ˂* 0.05) increased at 0–8 h and 0–24 h after treatment with MJ and MWCNTs, respectively. There was no significant (*P ˂* 0.05) difference in the MDA content between the leaves harvested at other times of exposure and those of control. The content of MDA reached the maximum values in the leaves exposed to MJ (10.18-fold higher than control) and MWCNTs (17.01-fold higher than control) at 2 and 8 h after exposure, respectively.

### Effect of MWCNTs and MJ on phenolic acids profiles

**Fig. 2:**
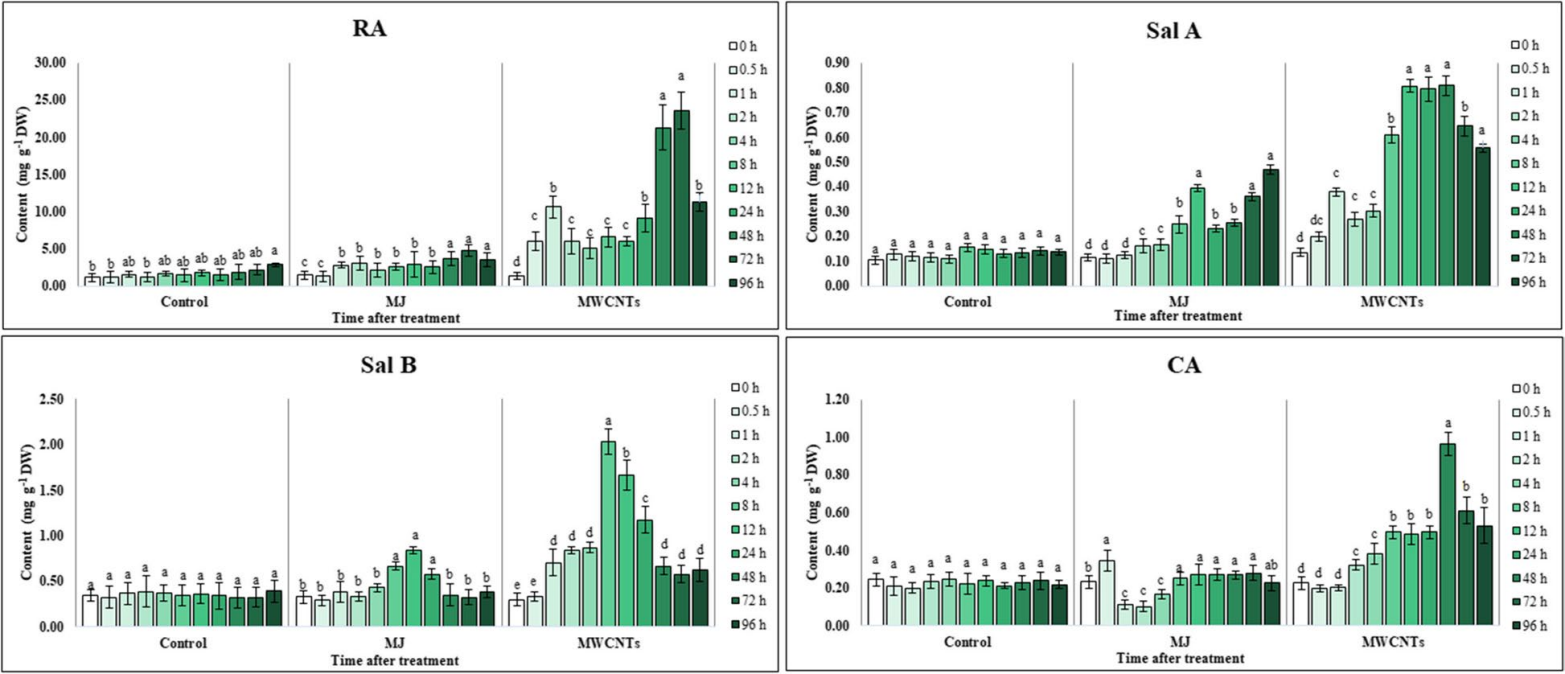
Changes in the phenolic acids content of the *S. verticillata* leaves after elicitation with MWCNTs and MJ. RA: rosmarinic acid, Sal B: salvianolic acid B, Sal A: salvianolic acid A, CA: caffeic acid. Bars not sharing the same superscript letter(s) are significantly different at *P <* 0.05 within each treatment group based on Duncan’s multiple range test. All data are means of three replicates, with error bars indicating SE

Based on the data obtained from HPLC chromatograms, four phenolic acids comprising RA, Sal A, Sal B, and CA were detected in the methanolic extract of all samples. We found that the production of these compounds significantly changed (*P <* 0.05) in the leaves with a different pattern at various harvesting times after exposure to MWCNTs and MJ (Fig. 2).

The RA content, as the most abundant phenolic acid, was increased (approximately 2-3-fold) during the 1 to 2 and 24 to 72 h intervals after treatment with MJ compared to the control. The highest content of RA was found at 2 and 72 h exposure of the leaves to MJ with values of 3.07 ± 0.97 and 4.78 ± 0.81 mg g^−1^ DW, respectively. While MWCNTs led to increases in RA production up to 11.52 times the control (23.63 ± 2.49 mg g^−1^ DW) 72 h after treatment. In addition, by the beginning of exposure to MWCNTs, RA content was considerably raised in the leaves by a value of 5.07 ± 1.33 mg g^-1^ DW to 21.32 ± 2.97 mg g^−1^ DW.

As a result, substantial increases were observed in the Sal A accumulation of the treated leaves with both elicitors compared to the control. The maximum value for Sal A content was achieved 96 h after elicitation with MJ (0.46 ± 0.03 mg g^−1^ DW) and 12 to 48 h after exposure to MWCNTs (0.79 ± 0.08 mg g^−1^ DW to 0.80 ± 0.04 mg g^−1^ DW) which was approximately 3.44- and 6.13-fold higher than control (0.13 ± 0.01 mg g^−1^ DW).

Similar to other phenolic acids, the content of Sal B significantly (*P ˂* 0.05) changed in the leaves after exposure to MJ and MWCNTs. However, in the first and the last h of harvesting after treatment with MJ, no significant (*P ˂* 0.05) difference was found in the content of Sal B between the exposed leaves and the control. Although, at times intervals of 4 to 48 h after treatment with MJ, detectable changes were observed in the content of Sal B in the exposed leaves compared to the control, and reached its maximum value (0.84 ± 0.07 mg g^−1^ DW), 8 h after treatment. The content of Sal B in the leaves was attained at its maximum level (2.03 ± 0.14 mg g^−1^ DW) after 8 h elicitation with MWCNTs, so that was 5.97-fold higher than the control (0.34 ± 0.09 mg g^−1^ DW). However, the content of this phenolic acid markedly declined at intervals of 12 to 96 h after exposure.

The pattern of changes in CA accumulation under MJ elicitation revealed that this phenolic acid was significantly (*P ˂* 0.05) increased by 0.5 h after exposure (0.21 ± 0.04 mg g^−1^ DW) and then decreased with time. Furthermore, the highest content of CA (0.96 ± 0.04 mg g^−1^ DW) was recorded in the leaves that were harvested 48 h after treatment with MWCNTs, 0.45-fold more than control (0.22 ± 0.04 mg g^−1^ DW).

### Effect of MWCNTs and MJ on expression profiles of *PAL, TAT*, and *RAS*

**Fig. 3:**
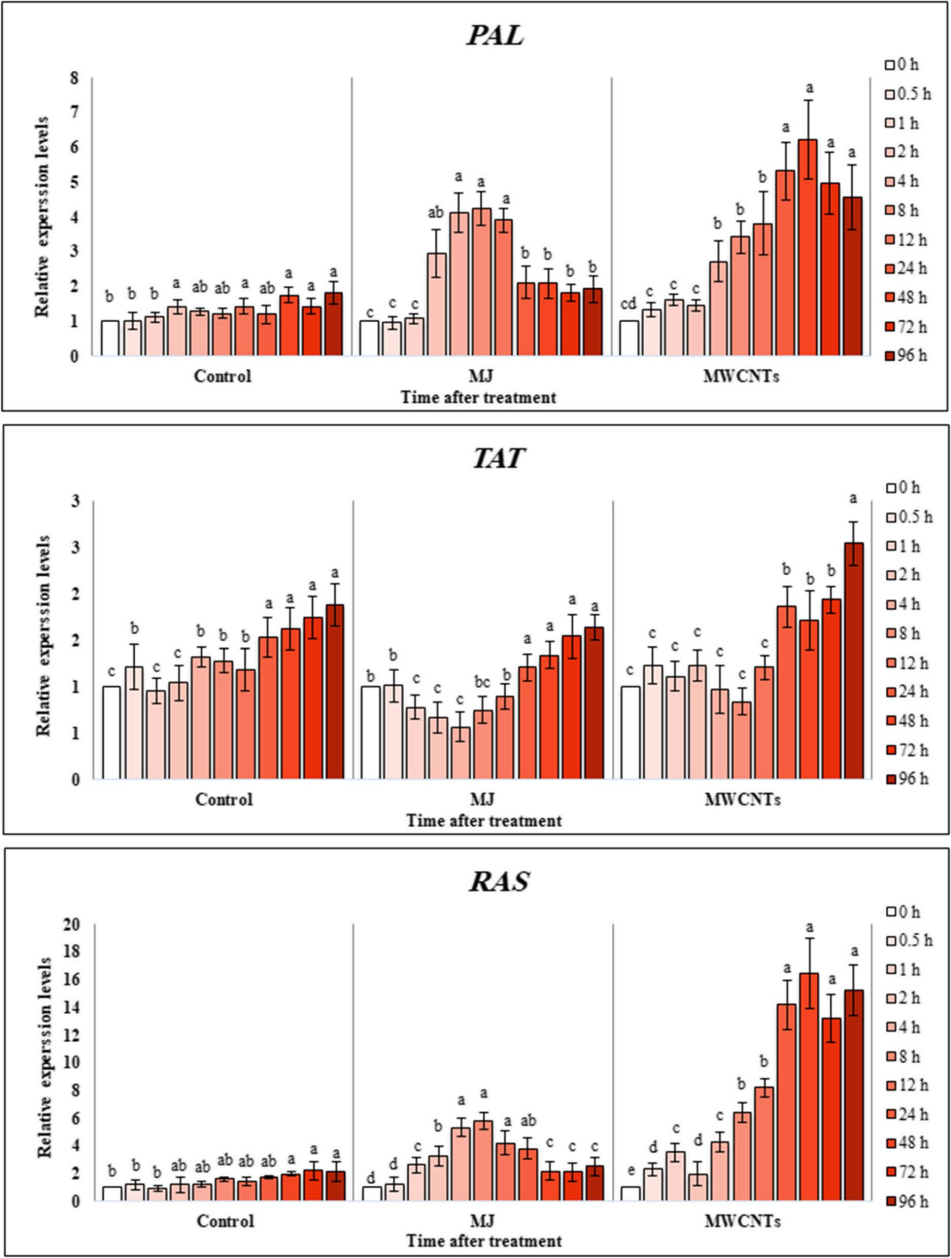
Changes in the *PAL*, *TAT*, and *RAS* relative expression levels of the *S. verticillata* leaves after elicitation with MWCNTs and MJ. *PAL*: phenylalanine ammonia-lyase, *TAT*: tyrosine aminotransferase, *RAS*: rosmarinic acid synthase. Bars not sharing the same superscript letter(s) are significantly different at *P <* 0.05 within each treatment group based on Duncan’s multiple range test. All data are means of three replicates, with error bars indicating SE

As seen in Fig. 3, transcription levels of three key enzymes involved in the phenolic acid biosynthesis pathway, including *PAL*, *TAT*, and *RAS*, were dramatically affected by exposure to MJ and MWCNTs in a time-dependent manner. The data obtained from the Real-time PCR analyses indicated that compared with control leaves, the expression level of *PAL* was increased 2 h after MJ elicitation (2.95-fold of control) and reached a maximum level after 8 h (3.41-fold of control), then dropped with the increase in exposure time. Similar to elicitation with MJ, an increasing trend, but with a temporary delay, in *PAL* expression level was observed in the exposed leaves to MWCNTs. Therefore, the highest gene expression level of *PAL* (4.45-fold of control) was detected 48 h after exposure to MWCNTs.

Compared to the control, the relative expression levels of *TAT* were significantly (*P ˂* 0.05) decreased in the harvested leaves after MJ elicitation. However, the expression levels of the gene declined initially at the beginning of the exposure times and subsequently rose after 96 h in the leaves treated with MWCNTs (1.35-fold of control). Changes in the transcript levels of *RAS* in the leaves, as the end-point enzyme in the RA biosynthetic pathway in response to elicitation with MJ and MWCNTs, were similar to that of *PAL* so that its expression levels increased with the increase in the time of exposure.

### Effect of MWCNTs and MJ on phytohormone profiles

**Fig. 4:**
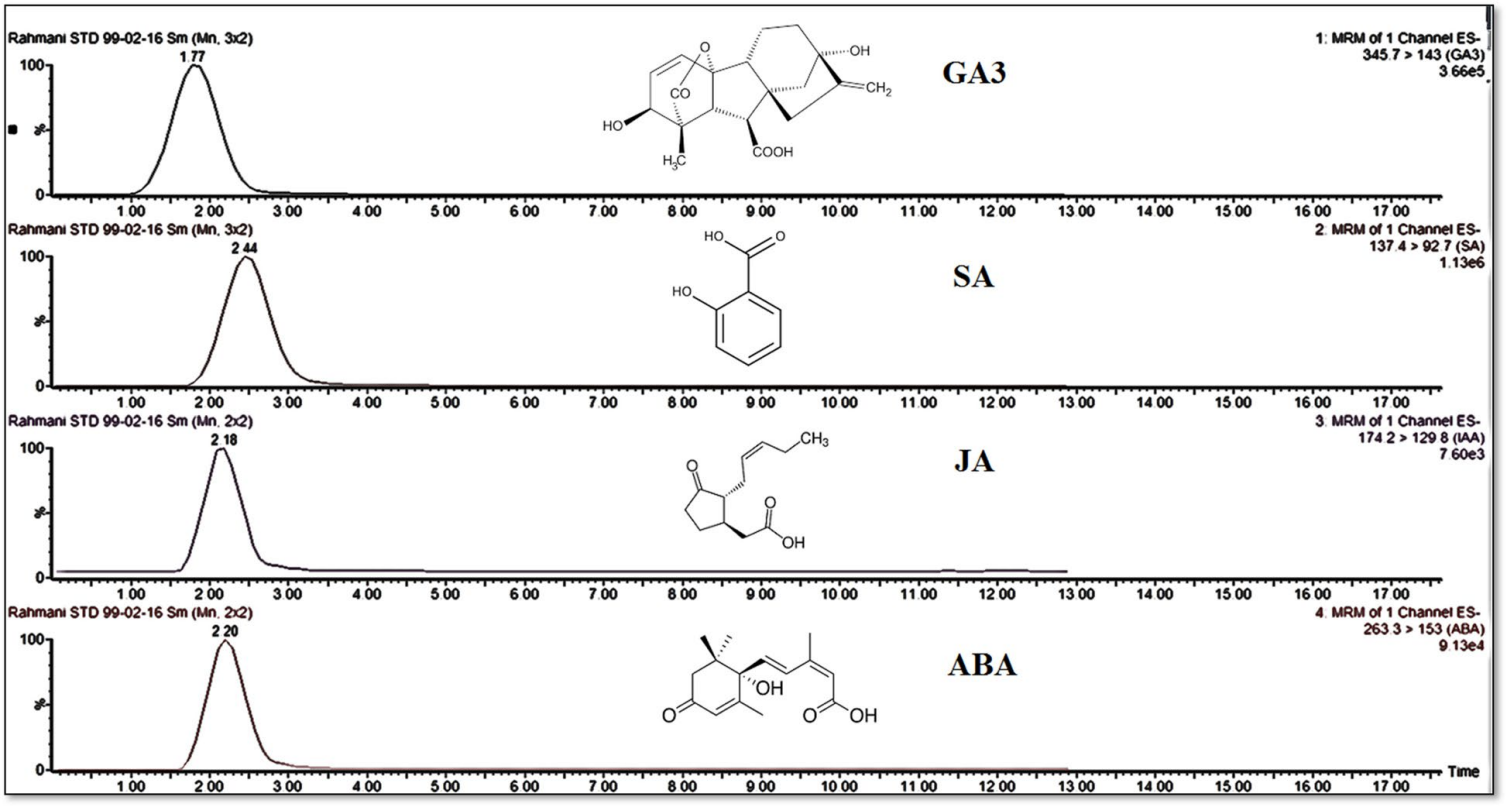
HPLC-ESI-MS/MS chromatograms of the studied phytohormones. GA_3_: gibberellic acid, SA: salicylic acid, ABA: abscisic acid, JA: jasmonic acid

**Fig. 5:**
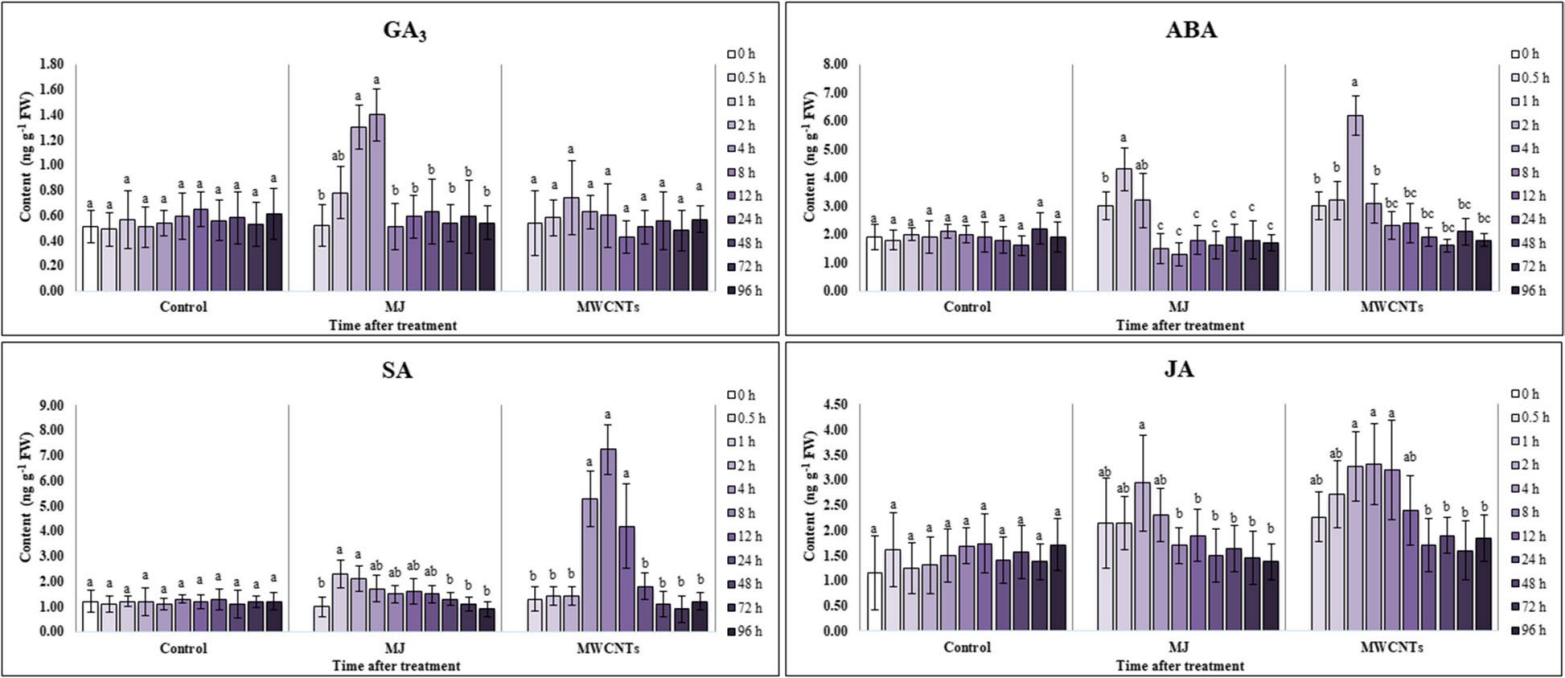
Changes in the concentration of phytohormones of the *S. verticillata* leaves after elicitation with MWCNTs and MJ. GA_3_: gibberellic acid, SA: salicylic acid, ABA: abscisic acid, JA: jasmonic acid. Bars not sharing the same superscript letter(s) are significantly different at *P <* 0.05 within each treatment group based on Duncan’s multiple range test. All data are means of three replicates, with error bars indicating SE

HPLC-ESI-MS/MS analysis was performed to identify and quantify four phytohormones (ABA, SA, JA, and GA_3_) in the extracts of MJ- and MWCNTs-exposed leaves (Fig. 4). Based on the obtained results, changes in the phytohormones concentrations have appeared earlier than other examined parameters in response to both of applied elicitors (Fig. 5). The concentration of ABA, as a first phytohormone was enhanced in the leaves after being treated with MJ and MWCNTs (30 min after exposure). The highest concentrations of ABA were recorded in the harvested leaves 1 and 2 h after exposure to MJ and MWCNTs, which were 2.26- and 3.06-fold more than the control, respectively. No significant (*P ˂* 0.05) changes were found in ABA concentrations of leaves after 12 to 96 h elicitation compared to the control group.

The accumulation pattern of GA_3_ in the leaf samples was partly similar to ABA, but elicitation with MJ was more effective than MWCNTs on hormone production. The concentration of GA_3_ reached its maximum levels in the leaves exposed for 2 and 4 h (1.32- and 4.01-fold of control) to MWCNTs and MJ, respectively.

According to the data, both elicitors significantly (*P ˂* 0.05) influenced the level of JA in the treated samples. The concentration of this hormone raised to 2 h after the elicitation (nearly 2-fold of control) in the MJ-exposed leaves and subsequently dropped as exposure time increased. Nevertheless, the trend period of increasing JA concentration was longer in MWCNTs-exposed leaves, so the highest level of JA was observed in the leaves 2 to 8 h after elicitation.

Our finding demonstrated that elicitation with MJ and MWCNTs significantly (*P ˂* 0.05) affected the levels of SA in the leaves. Since harvested leaves, 4 to 12 h after exposure to MWCNTs had the highest concentrations of SA, approximately 6.03-fold more than those reported for the control. However, the concentration of the hormone attained its maximum level 2 h after elicitation with MJ, about 2.02-fold higher than those of the non-elicited ones.

### Correlation analyses

**Fig. 6:**
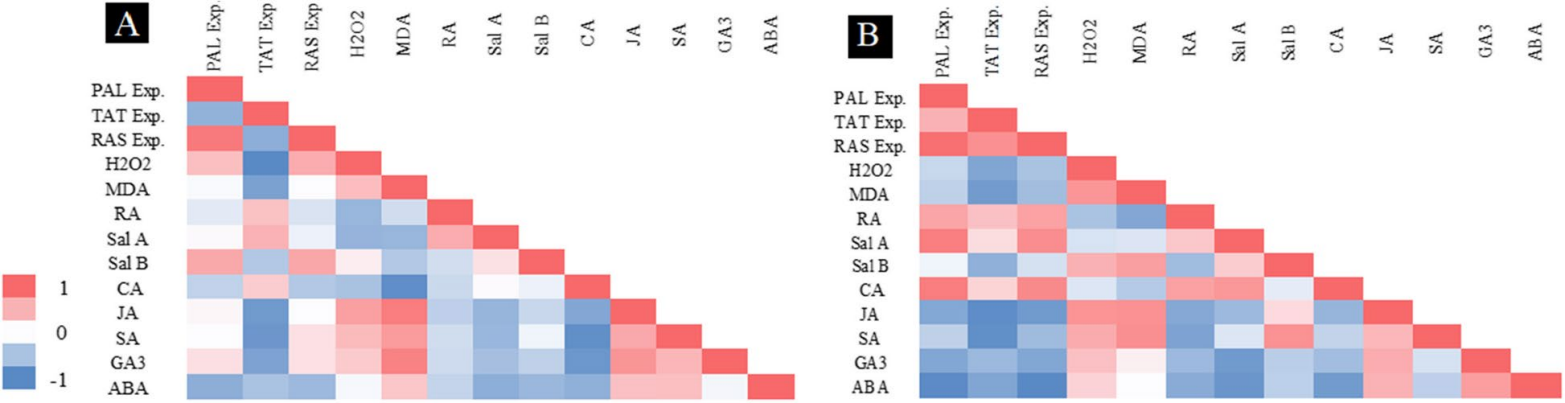
Heatmap correlations between all studied parameters in *S. verticillata* under elicitation with MJ (**A**) and MWCNTs (**B**). Pearson correlation coefficients were calculated and the statistical significance (*P ˂* 0.05) of correlations was determined. Red and blue regions show positive and negative effects, respectively. The color indication refers to the correlation coefficients. PAL Exp.: phenylalanine ammonia-lyase gene expression, TAT Exp.: tyrosine aminotransferase gene expression, RAS Exp.: rosmarinic acid synthase gene expression, MDA: malondialdehyde, H_2_O_2_: hydrogen peroxide, RA: rosmarinic acid, Sal B: salvianolic acid B, Sal A: salvianolic acid A, CA: caffeic acid, GA_3_: gibberellic acid, SA: salicylic acid, ABA: abscisic acid, JA: jasmonic acid

Heatmap of correlations between all measured parameters in the leaves of *S. verticillata* after exposure to MJ and MWCNTs is shown in Fig. 6. Obtained results from the data analyses of the MJ-treated leaves revealed substantial correlations between transcript levels of *PAL* with Sal B content (r_0.05_ = 0.683) and the content of Sal A with transcript levels of *TAT* (r_0.05_ = 0.637). Moreover, strong positive correlations were found between MDA content with JA (r_0.01_ = 0.906), GA_3_ (r_0.01_ = 0.875), and SA (r_0.01_ = 0.751) concentrations in the MJ-elicited leaves. Similar correlations were observed between the examined parameters for the MWCNTs-treated leaves, with some differences. As demonstrated in Fig. 6B, transcript levels of *PAL* and *RAS* displayed positive correlations with the content of phenolic acids, including Sal A (r_0.01_ = 0.902 and 0.836), CA (r_0.01_ = 0.985 and 0.850), and RA (r_0.01_ = 0.721 and 0.736) in the leaves after exposure to MWCNTs. In addition, statistical analyses revealed positive and significant correlations between JA concentrations with the contents of H_2_O_2_ (r_0.01_ = 0.800) and MDA (r_0.01_ = 0.814) in the MWCNTs-treated leaf samples. Moreover, changes in the concentrations of SA were linearly correlated with the contents of H_2_O_2_ (r_0.05_ = 0.695) and MDA (r_0.01_ = 0.824) in the treated leaves with MWCNTs.

**Fig. 7:**
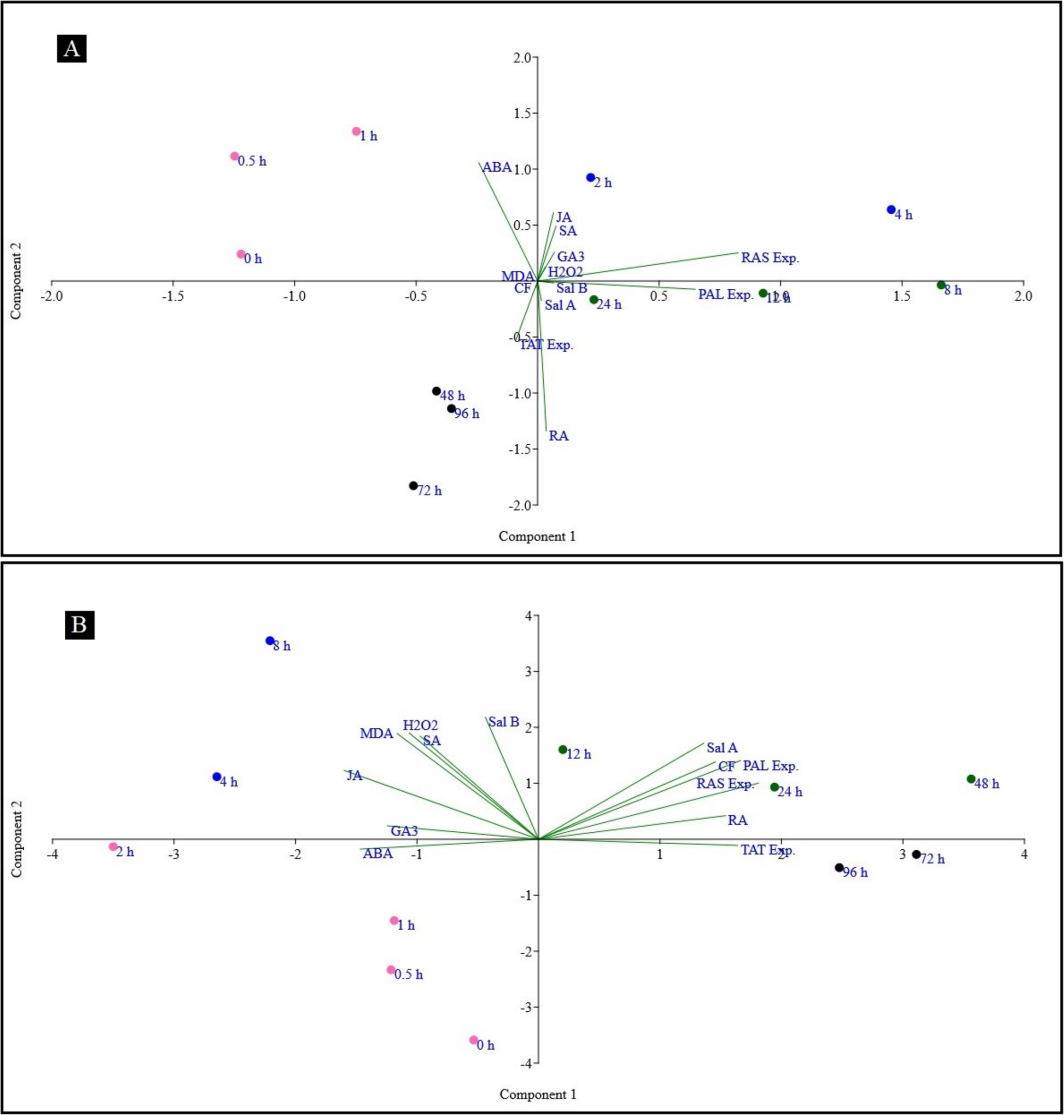
PCA biplot of the analyzed parameters in *S. verticillata* leaves after exposure to MJ (**A**) and MWCNTs (**B**). PAL Exp.: phenylalanine ammonia-lyase gene expression, TAT Exp.: tyrosine aminotransferase gene expression, RAS Exp.: rosmarinic acid synthase gene expression, MDA: malondialdehyde, H_2_O_2_: hydrogen peroxide, RA: rosmarinic acid, Sal B: salvianolic acid B, Sal A: salvianolic acid A, CA: caffeic acid, GA_3_: gibberellic acid, SA: salicylic acid, ABA: abscisic acid, JA: jasmonic acid

Results from principal component analysis (PCA) indicated considerable correlations between the tested parameters in the leaves after elicitation with MJ and MWCNTs (Fig. 7). According to the PCA biplot data, MJ- and MWCNTs-treated leaves were distributed into four groups. The leaves harvested 0 to 1 h after exposure to MJ were separated from others owing to the higher contents of MDA and ABA. Furthermore, the score for leaves that were 2 and 4 h under elicitation was mainly due to their higher contents of H_2_O_2_, JA, SA, and transcript level of *RAS*. The contents of phenolic acids (Sal A, Sal B, and RA) changed in the leaves harvested 8 to 24 h after exposure to MJ. Finally, leaf samples that were 24 to 96 h under elicitation correlated with higher transcription levels of *TAT* (Fig. 7A).

As shown in Fig. 7B, in the first group, the leaves elicited 0 to 2 h with MWCNTs separated from the other samples by having a higher ABA content. In the second group (4 to 8 h exposure), levels of phytohormones (GA_3_, JA, and SA), oxidative stress indices (H_2_O_2_ and MDA), and Sal B enhanced after elicitation. The leaves exposed 12 to 48 h to elicitor placed in the third group due to their higher contents of RA, Sal A, CA, and transcript levels of *PAL* and *RAS*. Lastly, the leaf samples exposed to MWCNTs for 72 to 96 h were separated into the fourth group because of the higher transcript levels of *TAT*.

## Discussion

When a plant responds to biotic and abiotic stresses, phytohormones organize signal transduction pathways and control all activities in the plant cells [53]. Concerning review in the literature, ABA, SA, and JA are crucial in how plants react to stressful situations. In addition, plant defensive reactions are controlled by crosstalk between PH signaling molecules. These interactions manage variations in gene expression and counteract the impacts of stress on plants [54].

After exposure to MJ and MWCNTs, we measured the changes in phytohormone concentrations in *S. verticillata* leaves. The obtained data revealed a rapid change in the levels of all examined phytohormones during the initial h after treatment. SA, ABA, and JA concentrations increased and peaked between 1 and 2 h after exposure of leaves to MJ and gradually decreased over time. Followed by, the highest concentration of GA_3_ was achieved within 2–4 h in the leaves after exposure to MJ. Similar to our finding, Zhao et al. [55] have suggested that using exogenous JA may enhance JA levels in *Panax quinquefolius* L. Additionally, Sun et al. [56] have shown that external stimuli can increase the production of salvianolic acids in *S. miltiorrhiza* by altering H_2_O_2_ concentration and JA levels during the early stages of treatment. These chemicals have been identified as the primary mediators in the signal transduction pathway that triggers phenolic acid synthesis.

Recent studies on signal transduction pathways revealed that the induction of the *COI1* gene led to the production of F-box proteins, which suppress the inhibitors of the JA signal transduction pathway. Additionally, the *JAR1* and *JIN1* genes, belonging to the MYC2 transcription factor family, have distinct roles in the JA signal transduction pathway; JAR1 is responsible for binding JA to isoleucine amino acid, while JIN1 regulates the transcription of JA-responsive genes. Another protein called JAZ mediates the communication between transcription factors and the COI1 protein. Ho et al. [57] documented these three proteins as the central core of the JA signal transduction pathway, which also facilitates JA interaction with other phytohormones signal transduction pathways. Zhou et al. [58] indicated that *Sm*MYC2a and *Sm*MYC2b are the key transcription factors responsible for the biosynthesis of phenolic acids in *S. miltiorrhiza*. In addition, they asserted that through interactions with inhibitory proteins *Sm*JAZ1 and *Sm*JAZ2, they control the gene expression of enzymes that participate in the biosynthesis of phenolic acids. RNA sequencing analyses of MJ-elicited *S. miltiorrhiza* by Ge et al. [59] showed that 25 out of 30 genes that play a role in the biosynthesis of phenolic acids were involved in signal transduction pathways of the hormone. They also introduced *Sm*JAZ_8_ as the main transcription factor that regulates the genes involved in the biosynthesis of phenolic compounds.

The obtained results confirm that ABA has a critical role in the production of phenolic acids after elicitation with MJ and MWCNTs. Similar to our results, studies have also shown that exogenous application of ABA enhanced the production of phenolic acids in *S. miltiorrhiza* by increasing the expression of genes involved in the biosynthesis of these metabolites, especially *PAL* and *TAT* [27, 60]. Recent findings verified the crucial role of the JAZ/MYC2 complex in the interaction between the JA and ABA signaling pathways and its regulatory function on similar responses of plants in various conditions. Consequently, the JA signal transduction pathway is linked by several molecules, including MAPK (Mitogen-activated protein kinase), GRX (Redox regulators of glutathione), and TRX (Thioredoxin) to the ABA receptor (PYL) [60].

In confirmation of our results, when the JA signaling pathway is stimulated, it can prevent the synthesis and accumulation of salicylic acid. These two hormones work together to affect various defensive responses in plants. Furthermore, SA can enhance a plant’s ability to withstand environmental stress, as shown in recent studies [57]. Externally applying SA has been found to increase PAL expression and cause RA accumulation in the aerial parts of *Salvia officinalis* L. and *S. virgata* [61]. In *Mentha piperita* L., exogenous SA has also been shown to promote the synthesis of phenolic compounds [62]. Similarly, co-treatment with SA and MJ has a more significant (*P ˂* 0.05) effect on the accumulation of phenolic compounds, particularly chlorogenic acid derivatives, in *Gardenia jasminoides* Ellis cell suspension cultures than either hormone alone [63].

According to our data, the changes in phytohormones observed in *S. verticillata* leaves differed after exposure to elicitors. We noticed that the stimulatory effect of MWCNTs on the production of JA was observed at longer times after treatment compared to MJ. This delay could be due to the absorption of MWCNTs by leaf epidermal cells and translocation. Although there was a minor increase in GA_3_ levels in treated leaves, it did not significantly (*P ˂* 0.05) affect the response of *S. verticillata* to MWCNTs elicitation. The concentration of phytohormones related to ABA and SA had the most significant (*P ˂* 0.05) changes, three and six times higher than controls, respectively. We found only one report on the impacts of carbon nanotubes on changes in phytohormone concentrations in rice seedlings grown hydroponically. The report showed that after 15 days of treatment, ABA, JA, GA_3_, and brassinolides concentrations decreased compared to the control group [64].

Our results suggested that changes in ABA and MJ concentrations, followed by an increase in SA content, caused a higher accumulation of phenolic compounds, especially RA, as a result of treatment with MWCNTs compared to MJ. The difference in GA_3_ levels between MJ and MWCNTs treatment groups could be attributed to the specific nature of the applied elicitors.

Studies have shown that MJ, like environmental elicitors, increases the production of ROS and disrupts cellular homeostasis. In these conditions, secondary oxidative stress is created and followed by stimulated defensive mechanisms in plants. Low concentrations of ROS act as a signaling molecule and control many different cellular pathways, while high concentrations are toxic and cause oxidative damage to plant cells. Therefore, maintaining ROS levels through activating enzymatic and non-enzymatic antioxidant mechanisms is essential to prevent oxidative stress damage [57]. In this study, changes in the oxidative stress indicator levels (H_2_O_2_ and MDA content) were investigated in the leaves of *S. verticillata* up to 96 h after treatment with MJ and MWCNTs. The results showed that H_2_O_2_ content and lipid peroxidation increased rapidly in the treated leaves when MJ reached its maximum values after eight h, then slowly decreased until they reached similar levels to the control group after 72 h. Similar to our results, an increase in MDA content due to lipid peroxidation following treatment with MJ was also reported in previous similar studies [57]. For example, MDA content was about 1.5 times higher than the control group in *Cnidium officinale* Makino treated with 200 µM MJ. In addition, Seo et al. [65] reported increased ROS content, especially H_2_O_2_, in the apoplast and symplast of *Helianthus annuus* L. roots treated with MJ. Equally, Farooq et al. [66] demonstrated that treating mustard leaves with MJ resulted in regulating transcriptional pathways related to the oxidative stress response. Consequently, the oxidative stress levels were reduced while the expression of genes encoding vital antioxidant enzymes, such as superoxide dismutase (SOD), peroxidase (POD), and catalase (CAT), was enhanced. After being stimulated with MJ, *Scrophularia striata* Boiss exhibited elevated levels of H_2_O_2_ content and enhanced activity in SOD, POD, and CAT [67]. MJ exposure led to an increase in the activity of antioxidant enzymes in *S. verticillata* leaves, as reported by Mazarei et al. [68]. Studies have indicated that the treatment of MJ can boost the efficiency of antioxidant enzymes, resulting in a decrease in the generation of ROS in plants exposed to different forms of biotic and abiotic stress [69]. This makes it a highly effective approach for boosting plant resilience to stress factors like drought, salinity, freezing, and herbivory [70, 71]. Some other studies have also reported increased ROS levels and antioxidant enzyme activities following treatment with nanoparticles in different plants [38, 72, 73].

Although the results obtained from the exposure of *S. verticillata* leaves to MWCNTs here were similar to those obtained for MJ, data showed that elicitation with MWCNTs induced more severe oxidative stress, leading to higher levels of lipid peroxidation and H_2_O_2_ content in leaves after treatment.

Our research shows a notable rise in the overall levels of phenolic acids, with an increase of up to twice the amount compared to the control plants, occurring between 12 and 72 h after exposure to MJ. The RA content in the leaves elicited by MJ showed an increase of up to 2.71 times in comparison to the control. After treating the leaves with MJ, the Sal A and Sal B contents showed a twofold increase within 12 h. Our results are in line with the findings presented by Zhou et al. [74]. Their study has demonstrated that treatment of *C. officinale* roots with 100 µM MJ has led to a twofold elevation in the accumulation of phenolic compounds compared to the control. In addition, they revealed that the rise in phenolic acid content was related to the increased activity of antioxidant enzymes. The study conducted by Attaran Dowom et al. [75] described the shoot culture of *Salvia virgata* Jacq. showed a significant (*P ˂* 0.05) increase in the content of phenolic acids, specifically RA and Sal A, after three days of exposure to a concentration of 11.2 ppm of MJ by up to two times compared to the control group. According to the findings of Ghasimi Hagh et al. [76], among the applied concentrations of MJ (50, 100, and 150 µM), 100 µM had the most significant (*P ˂* 0.05) effect in increasing the phenolic and flavonoid compounds (about three times that of the control plants) in *Salvia lerifolia* Benth. callus cultures. In addition, the content of RA increased by 1.5 times after treatment with MJ compared to the control.

By examining the effects of 50, 100, and 150 µM MJ on the callus cultures of *S. Khuzistanica*, Fatemi et al. [77] demonstrated that MJ at the concentration of 50 µM had the highest impact on the amount of RA (up to three times more than the control sample). Furthermore, Khojasteh et al. [78] have reported that treatment of *S. Khuzistanica* cells with 100 µM MJ increased the accumulation of RA up to 2.34 times compared to the control group. The increase in RA content was accompanied by increasing H_2_O_2_ content and the activity of antioxidant enzymes.

A literature review shows that in 60% of the research, MJ has been used as an effective stimulant to increase secondary metabolites with biological activities [57]. Our studies also revealed that the foliar spray of MJ enhanced the production of therapeutic compounds, especially RA, Sal A, and Sal B in *S. verticillata*.

Compared to leaves treated with MJ, those exposed to MWCNTs showed different patterns with various degrees of changes in the content of phenolic acids. Based on the findings, the sinusoidal changes pattern of RA content altered in response to MWCNTs. First, a two-fold rise in the accumulation of these compounds was seen during two h of treatment, after a minor decline, a considerable increase occurred in the content of this phenolic acid. As a result, the increase in RA content in the 72-h following treatment with MWCNTs was 11.52 times higher than that of the control. Additionally, compared to the leaves treated with MJ, the contents of Sal A and Sal B increased substantially more in the elicited samples with nanotubes. According to the literature, different nanoparticles could induce oxidative stress and stimulate the production of phenolic compounds in plants [19, 72, 79].

Based on the obtained data, the high level of oxidative stress produced by MWCNTs was responsible for the extent of the increase in the production of these compounds in the elicited leaves of *S. verticillata*.

In this study, gene expression patterns of the principal enzymes responsible for the biosynthesis pathways of phenolic acids were assessed in the elicited leaf samples. The results indicated that the transcript levels of two enzymes, *PAL* and *RAS*, exhibited the most significant (*P ˂* 0.05) changes. Furthermore, the study uncovered a strong and positive correlation between the levels of *RAS* transcripts and the content of phenolic acids in the leaf samples for both treated groups. This is in line with a study by Sadeghnezhad et al. [80] that an upsurge in flavonoids and phenolic acids production in the calli of *S. striata*, treated with 100 mM MJ, was linked to the modified expression levels of enzymes responsible for the synthesis of phenolic compounds, especially PAL. In addition, analysis of the gene expression of *PAL*, *TAT*, and *RAS* in the stimulated callus cultures of *S. Khuzistanica*, with different concentrations of MJ showed that the amount of RA accumulation was found to be directly related to a higher level of RAS expression [77]. In addition, Zhang et al. [81] demonstrated that MJ proved to enhance the production of RA, Sal B, and total phenol content in the cultivation of *S. miltiorrhiza* Bunge hairy root cultures but there were notable correlations found between the transcript levels of enzymes in the tyrosine-derived biosynthesis pathway and the contents of RA and Sal B compared to the phenylpropanoid pathway enzymes. The findings of Yu et al. [69] demonstrated that elicitation of *S. miltiorrhiza* hairy roots with MJ altered the expression of enzymes involved in the synthesis pathways of phenolic acids. Furthermore, they showed that the gene expression level of *Sm*PAL1 increased by around 14 times up to four h after treatment compared to control and then declined until 12 h after exposure. The alternation in gene expression patterns of *Sm*TAT1 and *Sm*RAS1 was comparable to that of *Sm*PAL1; however, the increases in the expression levels of *Sm*TAT1 and *Sm*RAS1 were notable and reached 25 and 16 times higher than the control, respectively. A study has shown that treating plants with MJ can lead to an increase in the activity of certain enzymes, such as PAL, which are involved in the biosynthesis of phenolic compounds [82]. Additionally, exposure to MWCNTs has increased gene expression related to the biosynthesis of various metabolites in *S. Khuzistanica* nodal segment cultures. In particular, RA accumulation was found to be 2.01-fold higher in MWCNTs-exposed cultures compared to the control [77].

Our research has shown that foliar spraying *S. verticillata* with MJ and MWCNTs can increase the concentration of phenolic acids by altering the gene expression levels of enzymes involved in their production pathways. Based on our findings, we believe that RAS is the most important enzyme in this species for RA synthesis.

## Conclusion

In this study, we observed significant (*P ˂* 0.05) variations in the phytohormone profiles of *S. verticillata* leaves following the perception of elicitors (MJ and MWCNTs), which could probably activate stress-response signal transduction pathways. It is to acknowledge that among the examined phytohormones, ABA functioned as a pioneer hormone and could establish a cooperative interaction between JA and SA within the elicited leaves. Simultaneously, as the levels of phytohormones changed, the contents of H_2_O_2_ and lipid peroxidation as oxidative stress indices increased in the treated leaves. Finally, the upregulation of enzymes involved in biosynthesis pathways led to significant (*P ˂* 0.05) accumulation in RA, Sal A, and Sal B contents of *S. verticillata* leaves after foliar exposure to MJ and MWCNTs. Obtained data indicated that MWCNTs were more effective than MJ in stimulating phenolic acid production in *S. verticillata* as a medicinal plant model. Therefore, the elicitation with MWCNTs can be a suitable practical tool for studying signal transduction pathways of secondary metabolites and improving their production in laboratory conditions.

## Data Availability

The raw data of this article will be made available by the corresponding author (Dr. Tayebeh Radjabian; rajabian@shahed.ac.ir), according to personal requests.

## Abbreviations

ABA: Abscisic acid
CA: Caffeic acid
GA_3_: Gibberellic acid
JA: Jasmonic acid
MDA: Malondialdehyde
MJ: Methyl jasmonate
MWCNTs: Multi-walled carbon nanotubes
PAL: Phenylalanine ammonia-lyase
PCA: Principal component analysis
RA: Rosmarinic acid
RAS: Rosmarinic acid synthase
ROS: Reactive oxygen species
SA: Salicylic acid
Sal A: Salvianolic acid A
Sal B: Salvianolic acid B
TAT: Tyrosine aminotransferase
